# Myocardial Work Assessment in Pediatric Subaortic Stenosis: Should the Doppler Gradient Be Added?

**DOI:** 10.1016/j.case.2026.07.004

**Published:** 2026-08-21

**Authors:** Michael Sammaan, Michael Moore, Suhair Shebani

**Affiliations:** East Midlands Congenital Heart Centre, Leicester, United Kingdom

**Keywords:** Myocardial work, Pressure-strain loop, Subaortic stenosis, LVOT obstruction, Pediatric cardiology, Echocardiography

## Abstract

• MW in LVOT obstruction depends on how LV pressure is estimated.
• Whether to add LVOT gradient remains methodologically unresolved.
• Gradient addition is physiologic but unvalidated in children.
• Adjusted methods increase MW indices versus standard approach.
• Consistent methods and clear reporting are essential in follow-up.

## Graphical abstract

**Figure:**
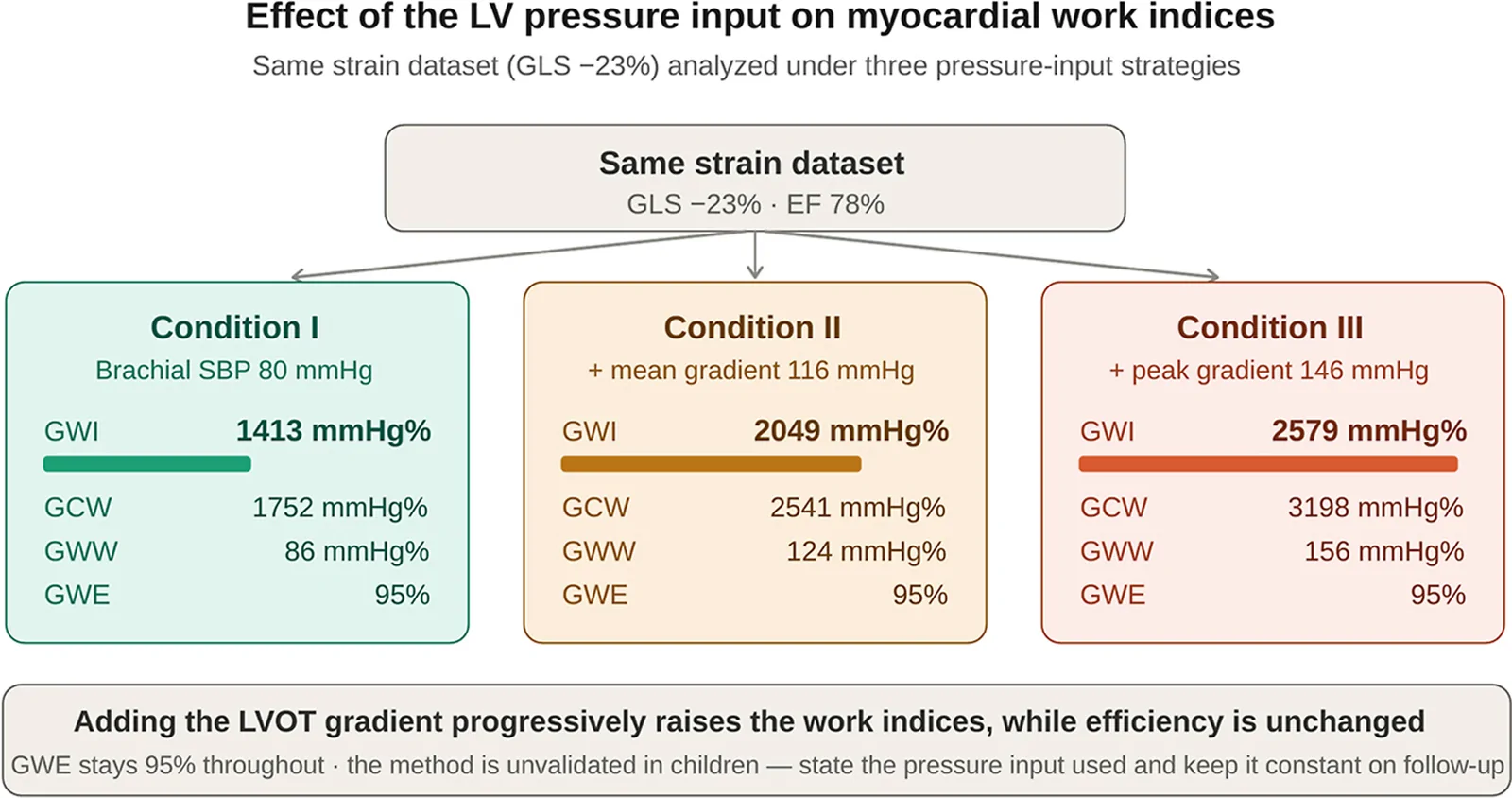

## Introduction

Noninvasive myocardial work (MW) analysis using pressure-strain loops has emerged as a novel echocardiographic tool for assessing left ventricular (LV) function.^1^, ^2^, ^3^ The method assumes that peak LV systolic pressure approximates brachial systolic blood pressure (BP)^4^; however, this assumption may not hold in the presence of left ventricular outflow tract (LVOT) obstruction, in which a pressure gradient exists between the LV and the systemic circulation.^5^

Whether the LVOT gradient should be incorporated into the estimated LV pressure curve remains controversial, particularly in pediatric patients, for whom validation data are limited. We present a 2-year 8-month-old patient with discrete subaortic stenosis and significant fixed LVOT obstruction, in whom MW assessment raised a practical methodological question. This case highlights the uncertainty surrounding LV pressure estimation in obstructive lesions and explores whether incorporation of the LVOT gradient into MW analysis is physiologically justified and clinically meaningful.^6^^,^^7^

## Case Presentation

A 2-year 8-month-old boy was referred from the general pediatric cardiology clinic to the advanced imaging clinic for further anatomical and functional assessment of progressive LVOT obstruction. The patient was initially referred to pediatric cardiology at 6 months of age after an incidental murmur was detected. The patient remained under general pediatric cardiology follow-up, during which a progressive increase in Doppler velocity across the LVOT was noted.

At the time of the advanced echocardiographic assessment, the patient weighed 12.8 kg and measured 93 cm, with a calculated body surface area (BSA) of 0.58 m^2^. The patient was awake and calm during image acquisition to minimize physiologic distortion of BP and MW assessment. Heart rate during the scan was relatively stable at ∼84 beats/min, although it could increase to ∼140 beats/min during agitation.

The electrocardiogram showed sinus rhythm with a normal frontal QRS axis and voltage criteria for LV hypertrophy, without abnormal repolarization changes. BP was measured at the beginning of the transthoracic echocardiogram (TTE) using an appropriately sized cuff on the right arm. The noninvasive right brachial BP was 80/40 mm Hg, and this value was used for MW analysis.

Comprehensive TTE demonstrated fixed subaortic stenosis due to a discrete subaortic membrane (Videos 1 and 2). There was no evidence of dynamic LVOT obstruction. The aortic valve was trileaflet with central coaptation, preserved mobility, no valvar aortic stenosis, and mild aortic regurgitation.

Pulsed-wave Doppler was used to localize the level of obstruction within the LVOT, and continuous-wave Doppler was used to measure the maximal velocity across the obstructed segment. The peak instantaneous LVOT velocity was 4.1 m/s (406 cm/s), corresponding to a peak instantaneous gradient of 66 mm Hg, with a mean gradient of 34 mm Hg (Figure 1).

**Figure 1:**
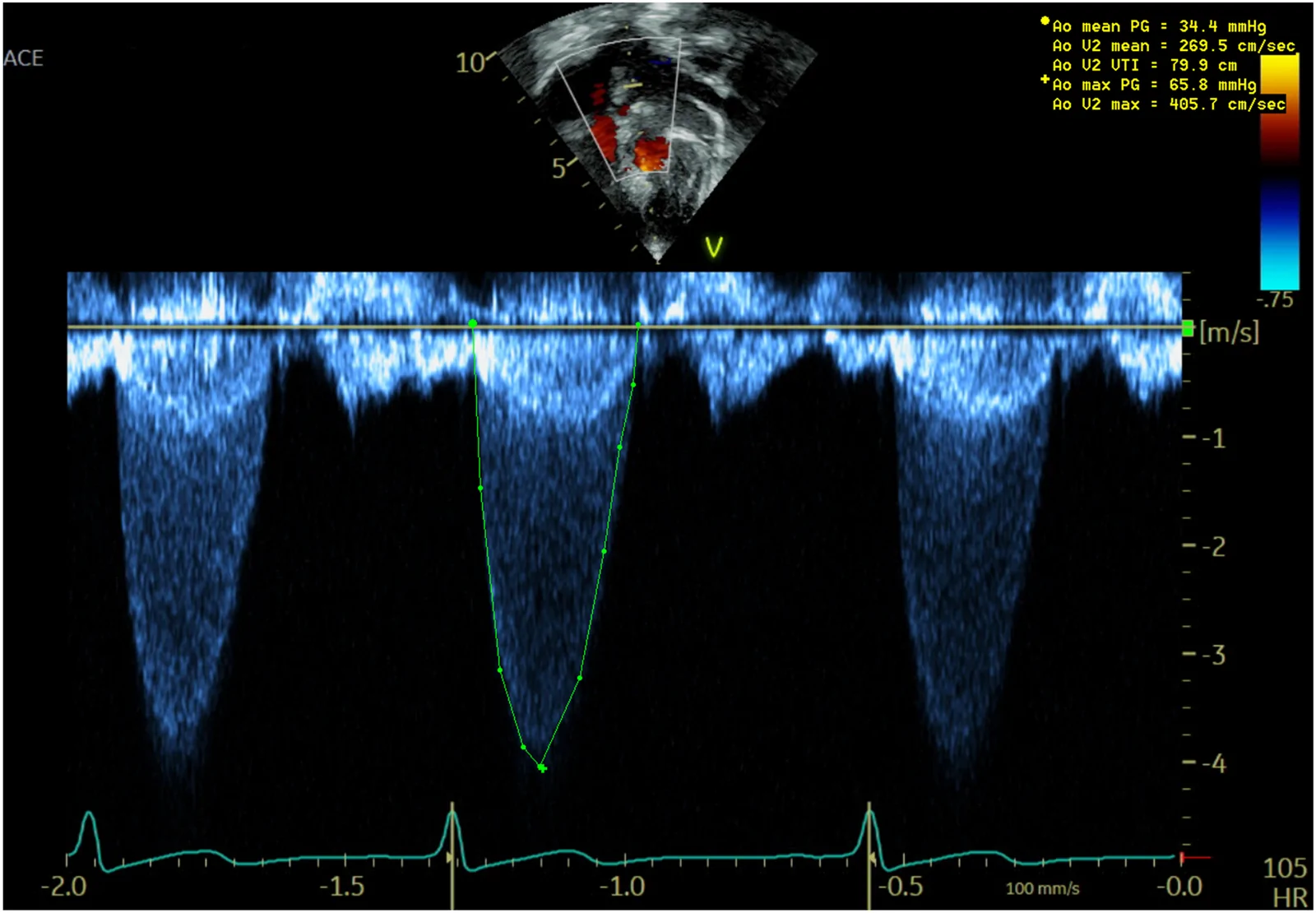
Transthoracic echocardiogram, apical 5-chamber, apex-down view with continuous-wave spectral Doppler display of the left ventricular outflow tract, demonstrates the high-velocity systolic envelope (4.1 m/s) at the subvalvular level and a parabolic fixed obstruction profile corresponding to a severely increased instantaneous gradient (peak, 66 mm Hg; mean 34 mm Hg).

LV volume and myocardial mass were estimated using the GE platform. LV systolic function was preserved and hyperdynamic, with an LV ejection fraction of 78%. The indexed LV end-diastolic volume was 95 mL/m^2^, corresponding to an absolute LV end-diastolic volume of ∼55 mL. The indexed LV end-systolic volume was 21 mL/m^2^, corresponding to an absolute LV end-systolic volume of ∼12 mL.

The indexed LV mass was 32 g/m^2^, corresponding to an estimated absolute LV mass of ∼19 g. This was above the 95th percentile for BSA and was followed as part of clinical monitoring of LV remodeling. LV wall thickness was mildly increased for BSA, with a Z score of approximately +1.9.

Global longitudinal strain was −23% (Figure 2A, Video 3). Left atrial strain was assessed to support interpretation of diastolic function. Left atrial reservoir, conduit, and contraction strain were 47%, 33%, and 12%, respectively.

**Figure 2:**
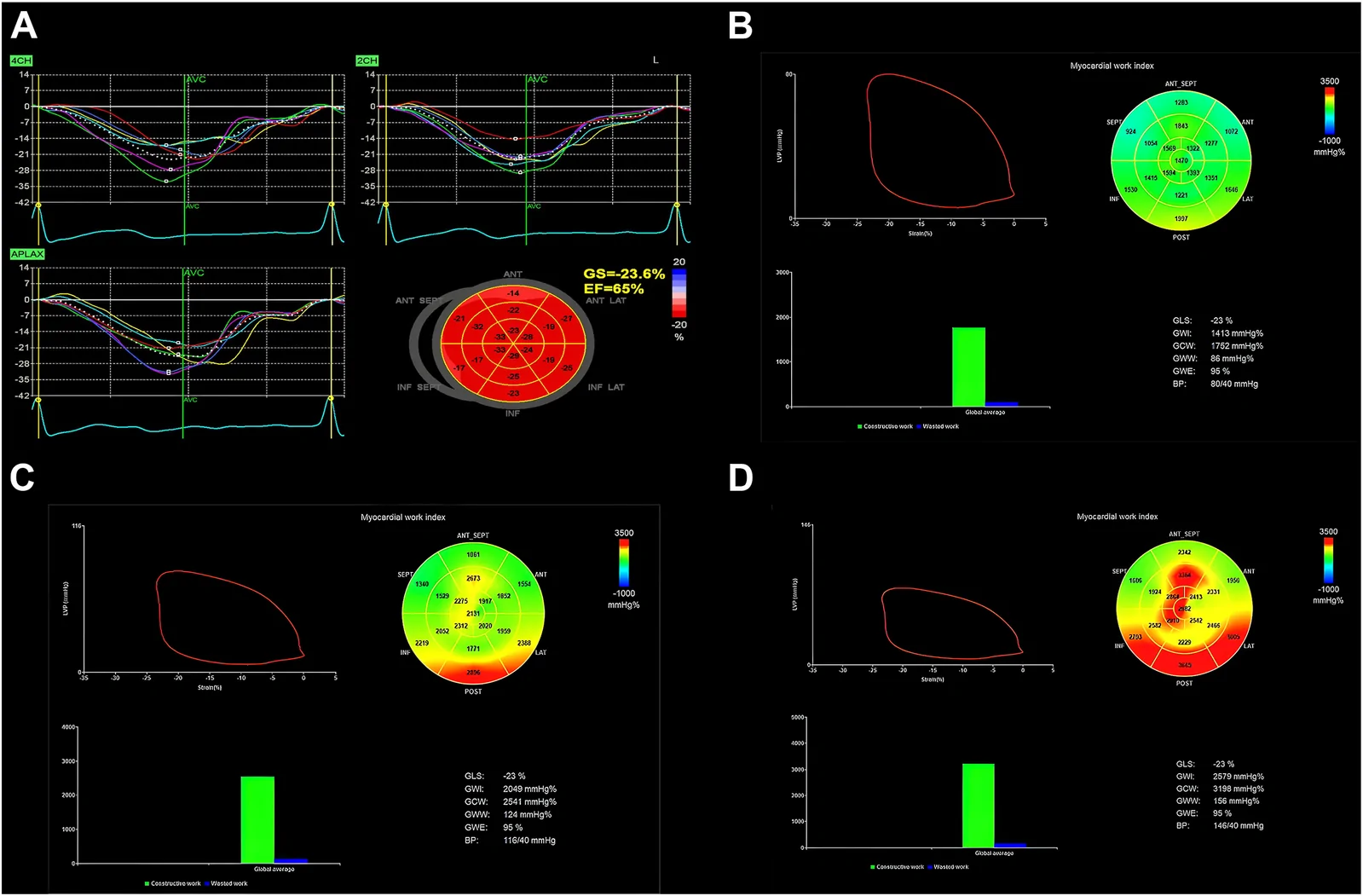
Myocardial deformation and MW analysis derived from a single speckle-tracking data set. (**A**) Segmental longitudinal strain from the 3 standard apical views with the corresponding bull's-eye map demonstrates a normal global longitudinal strain (−23%) and a normal global LV systolic function. (**B**–**D**) Noninvasive pressure-strain loop MW analysis of the same data set using the standard method of brachial systolic BP alone (**B**; 80/40 mm Hg; GWI 1413, GCW 1752, GWW 86 mm Hg%, GWE 95%), using the brachial systolic BP plus the mean LVOT gradient (**C**; 116/40 mm Hg; GWI 2049, GCW 2541, GWW 124 mm Hg%, GWE 95%), and using the brachial systolic BP plus the peak instantaneous LVOT gradient (**D**; 146/40 mm Hg; GWI 2579, GCW 3198, GWW 156 mm Hg%, GWE 95%). All 3 LV pressure input strategies display the pressure-strain loop, the segmental myocardial work bull’s-eye map with identical color scales, the constructive and wasted work bar graph, and the global indices. *EF*, Ejection fraction; *GS,* global strain; *GWI,* global work index*; GCW,* global constructive work*; GWW,* global wasted work*; GWE,* global work efficiency; *MW,* myocardial work*; LV,* left ventricular; *LVOT*, left ventricular outflow tract.

A small anterior mid-muscular ventricular septal defect (VSD) and a small restrictive patent ductus arteriosus were present. The VSD did not have a meaningful hemodynamic effect based on the available hemodynamic assessment.

MW analysis was performed using the GE Vivid Pioneer system with the EchoPAC MW plug-in. MW acquisition was incorporated into the same sequential segmental TTE as the anatomic and Doppler assessment. The LVOT Doppler gradient and MW assessment were obtained during the same study session. Two-dimensional image acquisition was optimized to achieve a frame rate greater than 60 frames/s. Timings of mitral and aortic valve event were automatically selected and were subsequently double-checked using tissue Doppler imaging at the septal mitral annulus.

Three pressure estimation strategies were used for MW analysis (Table 1): standard cuff BP alone, cuff BP plus the mean LVOT gradient, and cuff BP plus the peak instantaneous LVOT gradient. Progressive increases in calculated MW indices were observed when gradient correction was applied (Figure 2B–D).

**Table 1:** Myocardial work indices using different pressure-input strategies

	Condition I: Brachial systolic BP only	Condition II: Brachial systolic BP + mean LVOT gradient	Condition III: Brachial systolic BP + peak LVOT gradient
Rationale	Conventional MW methodology	Reflects the average systolic afterload	Approximates peak LV pressure
BP (mm Hg)	80/40	116/40	146/40
GLS (%)	−23	−23	−23
GWI (mm Hg%)	1413	2049	2579
GCW (mm Hg%)	1752	2541	3198
GWW (mm Hg%)	86	124	156
GWE (%)	95	95	95

*BP*, blood pressure; *LVOT*, left ventricular outflow tract; *GLS*, global longitudinal strain; *GWI*, global work index; *GCW*, global constructive work; *GWW*, global wasted work; *GWE*, global work efficiency.

The imaging findings were reviewed in a multidisciplinary team meeting to provide the surgical team with a clear anatomical and functional assessment of the LVOT obstruction. The echocardiographic assessment helped define the level of obstruction, confirm the fixed subaortic nature of the lesion, exclude dynamic obstruction and valvar aortic stenosis, and demonstrate the severity of obstruction using complementary anatomic, Doppler, strain, and MW parameters.

Given the significance of the fixed LVOT obstruction, the patient underwent subaortic membrane resection with a good surgical result and an uncomplicated short postoperative recovery. Predischarge TTE demonstrated preserved LV systolic function, no residual significant LVOT obstruction, and mild aortic regurgitation. The peak velocity across the LVOT was 1.2 m/s. The patent ductus arteriosus was ligated, and the small anterior mid-muscular VSD was left untreated.

The MW analysis was not used as the primary determinant of management. Rather, it was used as an illustrative and supportive functional assessment within the broader clinical and imaging evaluation.

## Discussion

This case illustrates an important methodological issue in noninvasive MW assessment in the setting of fixed LVOT obstruction. Standard MW analysis estimates LV pressure using brachial cuff systolic BP. In patients without obstruction, this approach provides a practical noninvasive approximation of peak LV systolic pressure. However, in fixed subaortic stenosis, LV systolic pressure may exceed peripheral systolic BP because of the pressure drop across the obstructed LVOT.

Adding the Doppler-derived LVOT gradient to the cuff systolic BP may appear physiologically reasonable because it may better approximate the pressure load experienced by the LV.^6^^,^^8^ However, this approach remains unvalidated, particularly in children.^2^^,^^9^^,^^10^ Doppler gradients are flow-dependent and may not fully represent the instantaneous pressure-strain relationship throughout systole. Pressure-strain loop algorithms reconstruct the LV pressure curve using an empirically derived reference waveform that is scaled in amplitude to the noninvasively estimated peak pressure and anchored in time to the valvular event timings, rather than being measured directly; they do not model the dynamic, flow-dependent transobstruction gradient, which itself varies through systole. Adding either the peak or the mean Doppler gradient as a single fixed offset to brachial systolic pressure, therefore, cannot reproduce the true time-varying LV pressure waveform and may misestimate instantaneous afterload at any given point in the cardiac cycle. In addition, MW algorithms were neither specifically designed nor validated for use in pediatric patients with congenital or acquired obstructive lesions.^4^

The distinction between fixed and dynamic obstruction is also important. In this patient, the obstruction was fixed and subaortic, without evidence of dynamic LVOT obstruction or valvar aortic stenosis. Therefore, the Doppler-derived LVOT gradient represented the pressure drop across a discrete subaortic membrane rather than a dynamic intraventricular gradient. Even in this case, adding a peak or mean Doppler gradient to brachial systolic BP remains a simplification of a time-varying pressure load.

This case also demonstrates why the selected pressure-input strategy should be explicitly reported. Using cuff BP alone produced lower MW indices than the mean-gradient-adjusted and peak-gradient-adjusted approaches. These differences may change how MW values are interpreted, particularly during serial follow-up. For this reason, consistency of methodology is essential when MW analysis is used in children with obstructive lesions.^9^^,^^11^

For pediatric echocardiographers, the practical message is that MW analysis in fixed LVOT obstruction should be interpreted cautiously and alongside conventional anatomic, Doppler, strain, and clinical assessment.^12^, ^13^, ^14^, ^15^ Cuff BP alone may underestimate LV pressure load, whereas gradient-adjusted methods, although mechanistically appealing, are not yet validated in this setting. Therefore, MW should be viewed as a complementary functional tool rather than a stand-alone basis for clinical decision-making in pediatric LVOT obstruction. In practical terms, the pressure input method used should be stated explicitly in the echocardiographic report and kept constant across serial studies in the same patient, so that changes in MW reflect physiology rather than methodology.

## Conclusion

MW assessment in pediatric LVOT obstruction remains methodologically challenging.^1^^,^^4^^,^^10^ This case shows that MW indices may vary substantially depending on whether the LVOT gradient is incorporated into LV pressure estimation. Transparent reporting of the pressure input strategy, cautious interpretation, and consistency across serial studies are essential until pediatric-specific validation data become available.^2^^,^^9^^,^^11^ This report is intended to stimulate discussion and awareness rather than propose a universal solution.

## Consent Statement

Written informed consent was obtained from the patient's parents for publication.

## Funding

The authors declare that this report did not receive any specific grant from funding agencies in the public, commercial, or not-for-profit sectors.

## Declaration of Generative AI and AI-Assisted Technologies in the Writing Process

During the preparation and revision of this work, the authors used generative AI-assisted tools (ChatGPT; OpenAI and Claude; Anthropic) to support language editing, structural organization, and formatting of the manuscript and to assist with the layout, labeling, and de-identification of the figures and supplementary videos and with the design of the schematic central illustration. All underlying echocardiographic images and measurements are the authors' own patient data and were not generated by AI. After using these tools, the authors reviewed and edited all content and take full responsibility for the content of the published article.

## Disclosure Statement

The authors report no conflict of interest.

## Supplementary Data

Video 1 Two-dimensional transthoracic echocardiogram, subcostal long-axis apex-down view without (A) and with (B) color-flow Doppler, demonstrates normal left ventricular (LV) size and function, LV hypertrophy, the discrete subaortic membrane with flow acceleration and aliasing, consistent with fixed subaortic obstruction, and mild aortic regurgitation.

Video 2 Two-dimensional transthoracic echocardiogram, parasternal long-axis focused view of the left ventricular outflow tract, blood speckle imaging acquisition, demonstrates the direction and acceleration of flow through the subaortic region (A); 3-dimensional parasternal view with simultaneous long-axis and short-axis (en face) 2-dimensional reference images (left) and volume-rendered reconstruction (right) demonstrates the discrete subaortic membrane (arrow).

Video 3 Two-dimensional transthoracic echocardiogram, apical 4-chamber apex-down view with speckle-tracking analysis, demonstrates the real-time tracking of the myocardial borders (A) and the segmental peak-systolic longitudinal strain with the corresponding strain curves and longitudinal strain map (B), consistent with normal, homogeneous deformation (global longitudinal strain, −23.2%).
